# The state of family medicine training programmes within the Primary Care and Family Medicine Education network

**DOI:** 10.4102/phcfm.v12i1.2588

**Published:** 2020-08-11

**Authors:** Klaus B. von Pressentin, Innocent Besigye, Robert Mash, Zelra Malan

**Affiliations:** 1 Division of Family Medicine, School of Public Health and Family Medicine, University of Cape Town, Cape Town, South Africa; 2 Department of Family Medicine, School of Medicine, College of Health Sciences, Makerere University, Kampala, Uganda; 3 Division of Family Medicine and Primary Care, Faculty of Medicine and Health Sciences, Stellenbosch University, Cape Town, South Africa

**Keywords:** family practice, primary health care, education, Health Workforce, family medicine, primary care, stages of change

## Abstract

The 2019 Primary Care and Family Medicine Education network (Primafamed) meeting in Kampala, Uganda, included a workshop that aimed to assess the state of postgraduate family medicine training programmes in the Primafamed network. Forty-six people from 14 African and five other countries were present. The evaluation of programmes or countries according to the stages of change model was compared to a previous assessment made 5 years ago. Most countries have remained at the same stage of change. Two countries appeared to have reversed their readiness to change as Rwanda moved from relapse to pre-contemplation and Mozambique moved from action to contemplation. Malawi, Zambia and Zimbabwe increased their readiness to change and moved from contemplation to action. Countries in the region remain quite diverse in terms of their commitment to family medicine training. Within Primafamed, it is possible for countries with a more advanced stage of change to assist countries with an earlier stage. Primafamed is also supported by a variety of partners outside of Africa. Five years after the previous country-level assessment, family medicine in Africa continues to span across all levels of the stages of change model. Stage-matched interventions aligned with the needs of individual countries should follow. Consequently, this workshop report will serve as a mandate and compass for Primafamed’s actions over the next few years, aimed at designing and delivering these interventions. As reiterated in the 2019 Kampala commitment, we should continue developing the discipline of family medicine (the medical ‘specialty’ of primary care), through alignment of our training programmes to the health needs in the African region.

## Introduction

Family medicine is making a valuable contribution to strengthening district health services in several countries in sub-Saharan Africa and has published a consensus statement on the role of the family physician.^1,2,3,4,5,6^ Although the roles of the family physician differ between countries and are still debated, it is clear that in Africa, the family physician often works at the district hospital as well as in primary care teams.^7,8,9^ Their roles are often different from that of family physicians in more highly resourced countries and they are seldom the first point of contact, which is usually with a nurse or clinical officer.^10^

Primary Care and Family Medicine Education network (Primafamed) is a network of academic departments of family medicine and primary care in sub-Saharan Africa (see: primafamed.sun.ac.za). Currently, it includes approximately 25 countries and 40 departments and has a history of building supportive south–south–north relationships aimed at developing family medicine training programmes and primary care research within its member institutions.^11,12,13^

The 2019 Primafamed meeting included a workshop that aimed to assess the state of postgraduate training programmes and to identify ways in which the network could be supportive. The workshop used the stages of change model (Table 1) as previously suggested by Stellenbosch University Rural Medical Education Partnership Initiative (SURMEPI) to categorise training programmes or countries.^14^ The stages of change model were originally used by Prochaska and Diclemente to describe intentional behavioural change at the level of an individual in the field of addictions.^15^ The stages of change have since become the central organising construct of the trans-theoretical model of change to explain behaviour change across a broad range of behaviours from the perspective of the individual to that of professional practices and organisations.^16^ Stellenbosch University Rural Medical Education Partnership Initiative has used this model to understand how policy-makers in key institutions change in their commitment towards implementing postgraduate training in family medicine.^14^

**TABLE 1:** Stages of change model as used in the workshop.

Stage	Description
Pre-contemplation	The country is not considering family medicine training in the near future.
Contemplation	The key actors (Ministry of Health who set human resources for health policy, universities or colleges who provide training and health professions council who regulate and register medical specialists) are considering the introduction of family medicine training but remain somewhat ambivalent.
Action	Postgraduate family medicine training has started, but there are no or few graduates, and family physicians are not yet an established part of the health system.
Maintenance	Training programmes are well established, and there is a consistent output of new family physicians who enter the health system.
Relapse	Although the country initiated postgraduate family medicine training, the programme disbanded or collapsed.
Permanent change	Training programmes are well established and integrated into the educational and health systems in such a way that relapse is now unlikely.

*Source:* Mash RJ, De Villiers MR, Moodley K, Nachega JB. Guiding the development of family medicine training in Africa through collaboration with the Medical Education Partnership Initiative. Acad Med. 2014;89(8):S73–S77. https://doi.org/10.1097/ACM.0000000000000328

## Workshop process

This 2-h workshop was part of a Primafamed meeting (4–5 June 2019) held before the 2019 WONCA Africa Conference in Kampala, Uganda. Forty-six people from 14 African and five other countries were present (Table 2).

**TABLE 2:** Countries represented in the workshop.

Country	Number of participants
Belgium	4
Brazil	1
Canada	6
Denmark	1
DR Congo	1
Kenya	5
Lesotho	1
Madagascar	1
Nigeria	8
Norway	1
Rwanda	1
Senegal	1
Sierra Leone	2
South Africa	6
Tunisia	1
Uganda	2
Zambia	1
Zimbabwe	2

The workshop commenced with a brief introduction to orientate the attendees to the stages of change model and group process. This was followed by a 45-min breakaway session in four groups: East African region, Francophone countries, West African region and Southern African region. The groups were asked to:

1. identify five strengths of their current training programmes
2. identify two or three issues or needs, which could be addressed by the Primafamed network
3. allocate each country or training programme to the most appropriate stage of change. The groups discussed the status of family medicine in their own countries as well as in other countries that they had information on in their respective regions.

After the group work, all attendees reconvened and gave a summary of their findings, which were discussed by the other participants.

## Workshop outputs

**FIGURE 1:**
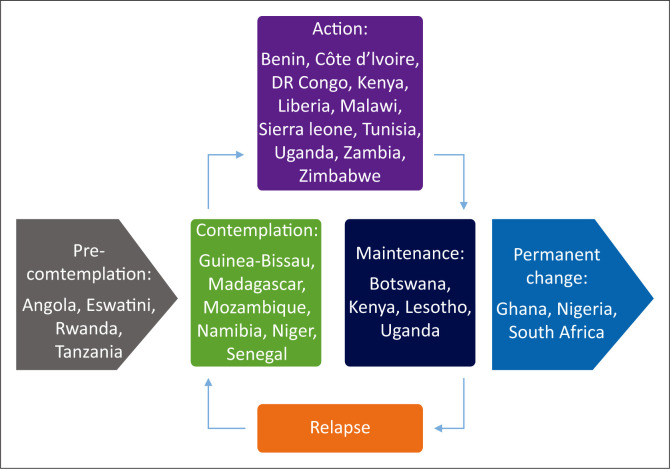
The stage of change of family medicine postgraduate training in selected African countries.

Figure 1 provides a graphical representation of how the groups allocated the countries according to the stages of change model.

### East African region

In Kenya, five higher education institutions offer family medicine training. Two of the institutions have established training programmes with a consistent output and were in the maintenance phase, whereas the three newer programmes were seen as still in the action stage. Postgraduate training was more established than undergraduate training, and programmes were located in both rural and urban areas as well as in the public and private sectors. The Ministry of Health is engaging with the discipline of family medicine, which facilitated a clearer job description as well as an increased recognition by the government. A national professional body for family medicine offers members support and enhanced communication through a WhatsApp group. This body has an opportunity to build consensus between the programmes on national learning outcomes, curricula, assessment standards and competencies required by family physicians. The group expressed an aspiration to anchor the focus of family medicine training more in the primary care setting.

In Uganda, the two programmes were in different stages: one programme was well established with a consistent output and the other programme was still struggling. Two out of 13 medical universities offer postgraduate family medicine training, whereas four offer undergraduate training. There is a need to standardise undergraduate family medicine curricula across the region. The policy environment for family medicine appears to be improving with more acceptance by government, salaries for family physicians on a par with other specialists, more opportunities for academic promotion and less ‘brain drain’ of family physicians to other countries.

In Rwanda, family medicine training was offered between 2008 and 2012, after which it was stopped. The attendees suggested that the country is now back to ‘pre-contemplation’ regarding family medicine training. Social and community medicine is offered to undergraduates. There exists a need for more advocacy to help persuade the government to recognise the discipline of family medicine.

In Tanzania, family medicine training is offered in the private sector in a network of health facilities attached to Aga Khan University. Government policy does not yet support family medicine in the public sector, and the country as a whole was rated as ‘pre-contemplative’. Accreditation of the college of family physicians by the central body is desired.^17^

### Francophone countries

There is no programme in Madagascar; however, the Ministry of Health is supportive and have asked for an investment in community-based family medicine. This country was rated as ‘contemplative’. There is a need to start training programmes, develop faculty and clarify the difference between general practitioners (GPs) and the proposed family medicine specialists.

Tunisia offers a 3-year programme with faculty development since 2011. Tunisia has a national college of family physicians, as well as four faculties of family medicine. The third cohort from the 3-year programme is about to graduate, and therefore, the country was rated as ‘action’ by the workshop attendees (but probably is meeting the definition of ‘maintenance’ with an output of family physicians over the past 3 years). More clarity is needed to distinguish GPs from family medicine specialists and a need for more family medicine research was expressed.

In Senegal, there is no separate family medicine programme and any training resides within the discipline of internal medicine. The Ministry of Health is ambivalent in terms of workforce planning, especially with regard to the distinction between GPs and family physicians. The attendees therefore rated the country as ‘contemplation’. There is a need for sharing of resources, such as family medicine modules during undergraduate training and to train clinical trainers.

One university (Université Protestante au Congo in Kinshasa) in the Democratic Republic of Congo (DRC) has established a department of family medicine. The development of this family medicine programme has been supported mainly by Sefako Makgatho Health Sciences University in South Africa. Thirty graduates from the 4-year programme are working in the country. They are experiencing challenges with retention, as the first cohort of graduates was lost to South Africa. Given the size of the country and scarcity of family physicians in the health system, the country was rated as ‘action’. There is a need for the training of clinical trainers, alignment of the curriculum with other countries and introduction of family medicine at an undergraduate level.

### West African region

The group contrasted the well-structured training in Nigeria to the early stage of the training in Sierra Leone, which was just accredited. There is an increase in family medicine departments, with a heightened awareness of family medicine as a specialty in West Africa. At policy level, the Nigerian Medical and Dental Council of Nigeria (MDCN) and the National Universities Commission (NUC) have developed Benchmark Minimum Academic Standards (BMAS), which include family medicine. Family medicine training also includes a diploma and additional degrees in lifestyle and geriatrics. There was a need for collaboration between countries to develop PhD programmes as well as the need for faculty development in areas of research, curriculum development and education. The self-rated stages of change differed within the region, with Nigeria and Ghana at ‘maintenance’; Sierra Leone, Benin, Liberia and Côte d’Ivoire at ‘action’; and Niger, Guinea-Bissau at ‘contemplation’.

### Southern African region

A key highlight is south–south collaboration. For example, the University of Stellenbosch in South Africa trained an initial nucleus of family physicians who spearheaded advocacy for and the initiation of family medicine training in Botswana and Zimbabwe. The strong support of the Primafamed network for family medicine education within the region was a strength. For example, Primafamed led a twinning programme, whereby each programme in South Africa twinned with an emerging programme in another country. There is a desire to share resources and curricula such as the diploma curriculum, e-learning resources and the portfolio for workplace-based learning and assessment. Many tools developed for postgraduate education may also be useful to undergraduate curricula.

There is increased credibility and improved rigour in the assessment of training programmes via partnerships. In South Africa, for example, the validity and reliability of the national licencing examination improved through a partnership between the Royal College of General Practitioners (RCGP) and the College of Family Physicians. External examiners help with improving the standard of examination, but it is difficult for programmes with small numbers of faculty and students and limited expertise in assessment to develop national examinations at the appropriate standard. The idea of a combined eastern, central and southern African college of family physicians is being explored as a way of combining resources and expertise in assessment.

Another strength is the Training of Clinical Trainers faculty development initiative offered in partnership with the RCGP.^18,19^ Specialists from other disciplines also attended the training, which gave them better insight into family medicine training. There is a need to train more clinical trainers and to support ongoing formative assessment visits to improve the learning environments.^20^

In South Africa, there exists a consensus at national level regarding the training outcomes, skills list, curriculum content and the national college exit examination.^21^ A National Education and Training Committee maintains this consensus with all nine programmes and operates under the auspices of a national professional body, the South African Academy of Family Physicians.

Increasingly, research from the Southern African region is published in the *African Journal of Primary Health Care and Family Medicine* as well as the *South African Family Practice Journal*, which provide an evidence base for family medicine development and advocacy in the region.^22^

Eswatini and Angola were rated as ‘pre-contemplation’ because family medicine is not being discussed by the key stakeholders. Mozambique and Namibia were rated as ‘contemplation’, and in Namibia, family medicine training is already offered at diploma and undergraduate levels.^23^

Zambia was rated as ‘action’, with two registrars enrolled at the University of Zambia. Zimbabwe is also at ‘action’. At National University of Science and Technology (NUST) in Bulawayo, a programme started in 2020, although it remains fragile and under-resourced. Training at the University of Zimbabwe is also expected to start soon. Despite instability at a political level, there seems to be good support from the Ministry of Health. Malawi was rated at ‘action’ as there are no graduates yet, but this was also expected to happen soon.

Lesotho was rated at ‘maintenance’ as there is an established programme. However, there is still a risk of relapse as the current programme relies on external support from Boston and the University of the Free State, and the career path for family physicians is not yet defined. Botswana is at ‘maintenance’, as the programme has produced graduates who are now entering the health system.

South Africa is at ‘permanent change’. There are nine training programmes with a consistent output and substantial numbers of family physicians employed in the public and private sectors. However, there is a challenge with the availability of more posts and the role of family physicians in the primary health care setting is not yet clear within national policy.

## Discussion

The evaluation of programmes or countries according to the stages of change model can be compared to the assessment made by SURMEPI 5-years ago.^14^ Most countries have remained at the same stage of change. Two countries appeared to have reversed their readiness to change as Rwanda moved from relapse to pre-contemplation and Mozambique moved from action to contemplation. Malawi, Zambia and Zimbabwe increased their readiness to change and moved from contemplation to action. Countries in the region remain quite diverse in terms of their commitment to family medicine training.

Within Primafamed, it is possible for countries with a more advanced stage of change to assist countries with an earlier stage. However, it should be noted that the purpose of identifying what stage each country is at is to mould the intervention to the stage. Stage-matched interventions can have a far greater impact than one-size-fits-all programmes by increasing the likelihood of action.^16^ In addition, countries who share the same stage may also require individualised interventions to prevent resistance to change in their respective contexts.

The Primary Care and Family Medicine Education network is also supported by a variety of partners outside of Africa, particularly the University of Ghent. Specific examples of how the network can assist were identified:

- Generate research evidence of the contribution of family medicine to health systems and the role of family physicians in our context, as well as evidence that supports the development of family medicine education (such activities may include assisting with conducting research and publishing findings on the impact of family medicine in the respective African countries).
- Assist with advocacy for the discipline of family medicine by sharing experiences and evidence between countries. Explaining why postgraduate training is required and how family physicians differ from GPs was a cross-cutting theme.
- Share curricula and educational resources such as books, e-learning materials and portfolios.
- Provide external assessment and feedback to improve the quality of examination.
- Provide opportunities for faculty development, including clinical trainers, and in some cases share faculty members to assist with emerging programmes.

## Conclusion and way forward

Five years after the previous country-level assessment, family medicine in Africa continues to span across all levels of the stages of change model. Stage-matched interventions aligned with the needs of individual countries should follow. Consequently, this workshop report will serve as a mandate and compass for Primafamed’s actions over the next few years, aimed at designing and delivering these interventions. As reiterated in the 2019 Kampala commitment,^24^ African family doctors are actively exploring how we can contribute to strong primary health care teams through expanding family medicine training and building the capability of all team members. This necessitates that we should continue developing the discipline of family medicine (the medical ‘specialty’ of primary care), through alignment of our training programmes to the health needs in the African region.
